# Efficacy of rituximab in thymoma associated minimal change disease: case report

**DOI:** 10.1186/s12882-021-02479-8

**Published:** 2021-09-07

**Authors:** Zhour El Ouafi, Clovis Mugnier, Robin Jeannet, Clément Danthu, Marion Duval, Valère Belle Mbou, Fatouma Touré

**Affiliations:** 1 CHU Limoges, Division of Nephrology, Limoges, France; 2 CHU Limoges, Division of Immunology, Limoges, France; 3 CNRS 7276 - Inserm U1262, CRIBL, University of Limoges, Limoges, France; 4 CHU Limoges, Division of Anatomopathology, Limoges, France

**Keywords:** Glomerulonephritis, Minimal change disease, Thymoma, Rituximab

## Abstract

**Background:**

Thymomas have been associated with a broad spectrum of autoimmune diseases. Minimal change disease (MCD) is the most frequent pathological lesion reported. Pathophysiological mechanisms involved in secondary MCD, and linking MCD to thymoma are not yet fully explained, although the hypothesis of T cell dysfunction has been suggested. The fundamental therapeutic principles are steroids and surgical treatment of thymoma, but failures and relapses often require immunosuppressant combinations.

**Case presentation:**

A 62-year-old female was admitted in our unit for a nephrotic syndrome associated with a thymoma. The diagnosis of thymoma associated MCD was confirmed by kidney biopsy. After surgical resection of the thymoma and steroid therapy, no remission was observed. Immunosuppressive therapy was then intensified with introduction of rituximab. Here, we report a steroid-resistant nephrotic syndrome secondary to MCD associated thymoma, which achieved complete remission after rituximab therapy. To the best of our knowledge, this is the first report of the use and efficacy of rituximab therapy in this pathology.

**Conclusions:**

Our case report suggests that primary and secondary MCD may share similar pathophysiological mechanisms. It does not allow us to draw any conclusions about the mechanism of action of rituximab, but we believe this report argues for the safety and efficacy of rituximab use in thymoma-associated MCD, and therefore constitutes a rationale for future studies.

## Background

Thymomas have been associated with a broad spectrum of autoimmune diseases. Renal involvement has also been described in patients with thymic tumors, but this association is rare. Minimal change disease (MCD) is the most frequent pathological lesion reported. The pathophysiological mechanisms involved in primary idiopathic MCD or secondary MCD, and linking MCD to thymoma, are not yet fully explained, although the hypothesis of T cell dysfunction has been suggested. The fundamental therapeutic principles are steroids and surgical treatment of thymoma, but failures and relapses often require immunosuppressant combinations. Here, we report a case of steroid-resistant nephrotic syndrome secondary to MCD associated thymoma, which achieved complete remission after rituximab therapy.

## Case presentation

**Fig. 1:**
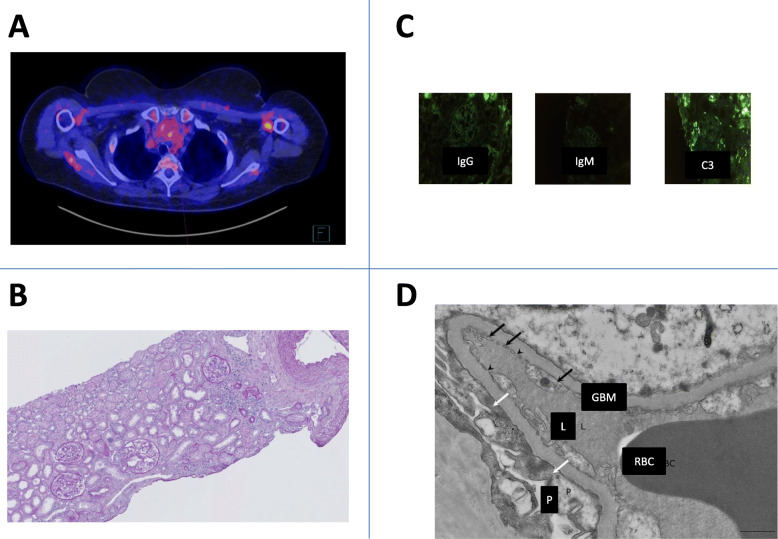
Images of the thymoma and renal histology. **A** Transaxial Fluorine-18 Fluorodeoxyglucose positron emission tomography - computed tomography (F-18-FDG PET/CT) images show a nodular thymic mass in the anterior mediastinum with moderate uptake (maximum standardized uptake value = 4). **B** Light microscopy analysis (periodic acid-Schiff stain, × 100): the glomeruli show no proliferation, no sclerosis, no inflammation or necrosis and the Bowman space is unremarkable. **C** Immunofluorescence microscopy found no deposits (IgM, IgG, C3). **D** Ultrastructural analysis: transmission electron micrograph (JEOL JEM 1400 Flash) of peripheral capillary loops demonstrating extensive foot process effacement, with no immune complexes, normal mesangial cellularity and matrix, and normal thickness of the glomerular basement membrane There is no evidence of podocyte detachment consistent with a diagnosis of FSGS. Black arrow: endothelial cell. Arrowhead: fenestrae. White arrow: diffuse effacement of podocyte foot processes. (GBM: glomerular basement membrane. L: lumen of glomerular capillary. P: podocyte. RBC: red blood cell)

A 62-year-old female was admitted for dyspnea. Her only medical history was cutaneous lupus treated with hydroxychloroquine sulfate for 8 years. Physical examination found dyspnea, edema limited to the lower extremities and superior vena cava syndrome. Laboratory tests confirmed hypoxemia with PaO2 at 70 mmHg; and normal renal function (creatinine: 0.97 mg/dL). A computed tomography (CT) scan found normal pulmonary parenchyma and vascularization, but revealed the presence of an anterior mediastinal mass. A PET scan confirmed positive metabolic activity of the mediastinal mass (Fig. 1A). A biopsy was performed under mediastinoscopy. Pathological examination of the mass revealed the presence of pseudo-epithelial tumor cells of the thymus, while the thymic cortex was normal, without lymphocyte infiltration in the tumor tissue. These features led to a diagnosis of type A non-invasive thymoma (Fig. 1B).

While investigations on the mediastinal mass were being performed, the patient developed acute kidney injury (Creatinine 4.7 mg/dL), with nephrotic syndrome: serum albumin was 9.7 g/L, proteinuria was 17 g/g (composed of more than 80% of albumin) and microscopic hematuria was positive. In agreement with the nephrotic syndrome, a high level of cholesterol (total cholesterol = 3.07 g/l triglycerides = 1.19 g/l, LDL = 2.13 g/l), and lower levels of serum immunoglobulins (IgG 2.58 g/l, IgA 1.73 g/l, IgM 2.5 g/l) were also found. Results of the renal biopsy were as follows: normal kidney parenchyma on optical microscopy and immuno-fluorescence analysis without any proliferation or deposition. Electronic microscopy showed extensive foot process effacement corresponding to a loss of cell differentiation, loss of pedicels and increased collagen matrix production, creating a gap between the podocyte and glomerular basement membrane (Fig. 1D). No podocyte detachment was observed and there was no evidence in favour of a diagnosis of focal segmental glomerulosclerosis. Regarding immunologic tests, anti-nucleic antibodies were positive (*1/160),* but Extractable Nuclear Antigen (ENA) antibodies, complement fractions C3/C4, and anti-neutrophil cytoplasmic antibodies were negative. Similarly, tests for HIV, hepatitis B and C, and cryoprotein were all negative. Our final diagnosis was minimal change disease (MCD) associated with thymoma. Therefore, oral prednisolone (1 mg/kg/day) was initiated immediately and thymectomy was performed.

**Fig. 2:**
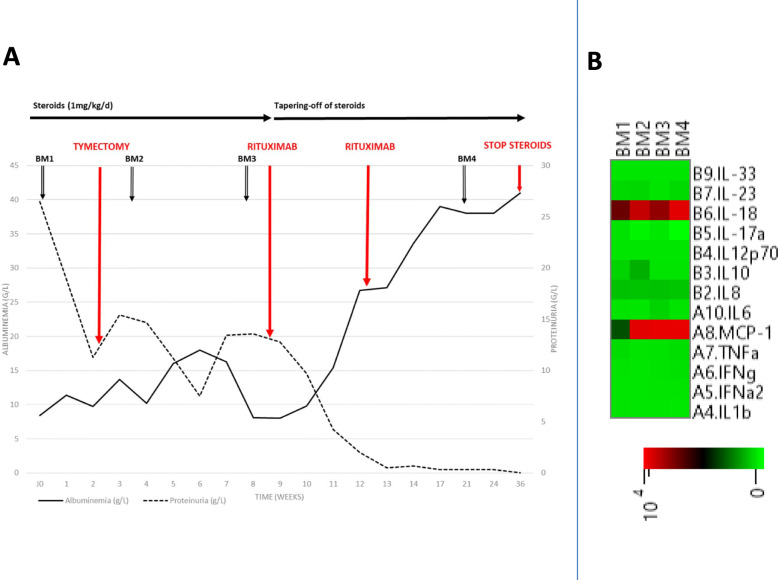
Outcome after treatment. **A** Trends in proteinuria and albuminemia after treatment with oral streroids, thymomectomy and Rituximab. **B** Cytokine profile in peripheral blood before and after treatment

Eight weeks after initiation of the steroid therapy, the patient showed a significant lack of response to steroid therapy, with serum albumin of 10 g/L, and proteinuria of 13.5 g/g, Clinical examination found exacerbation of asthenia as well as edema. Given the intensity and the persistence of nephrotic syndrome, with no improvement after steroid induction, we decided to reinforce the treatment with rituximab (1 g given twice, 15 days apart). We monitored the CD19 count (96/mm3 before administration and 0/mm3 after the second dose - this is in agreement with the profound lymphocyte depletion expected). Over the following days, we noticed a progressive improvement in both albuminemia and proteinuria. Complete remission was observed 3 weeks after infusion of rituximab. We followed French guidelines for tapering off the corticosteroids. After 11 weeks at high dose (1 mg/kg/day = 80 mg/d from 09/06/19 to 22/08/19), we pursued with 50 mg/d for 2 weeks, 35 mg/d for 2 weeks, 25 mg/d for 2 weeks, 20 mg/d for 2 weeks, 17.5 mg/d for 2 weeks, 15 mg/d for 2 weeks, 10 mg/d for 2 weeks, 7.5 mg/d for 4 weeks, 5 mg/d for 4 weeks, followed by steroid withdrawal using hydrocortisone. At the last follow-up, serum albumin was 38 g/L, proteinuria was 0.6 g/g and creatinine 0.83 mg/dL (Fig. 2A). The patient consented to collection of serum at different timepoints. We performed a cytokine profile using a bead assay (Legendplex human inflammation panel, Biolegend, San Diego, CA) on serum collected before (BM1, BM2 and BM3) and after treatment with rituximab (BM4). We did not find any deregulation of the cytokine profile before treatment with rituximab, either on the TH1 side (INFg, TNF, IL6, IL12) or the TH2 side (IL4, IL5, IL10, IL13). Cytokines were in the normal range, except for MCP1 and IL18, which were increased. Interestingly, no modification was found on the serum collected after treatment with rituximab, suggesting that the beneficial effect of this treatment was not mediated by a modification of the cytokine balance (Fig. 2B and C).

## Discussion and conclusion

To the best of our knowledge, the present case is the first to report the efficacy of rituximab in thymoma-associated MCD. This syndrome is considered to be sensitive to surgical removal of the thymoma and to steroid therapy, but we observed only a partial effect with this strategy in the present case. Reinforcement of the treatment with rituximab appeared to be beneficial and safe.

The thymus is a primary lymphoid organ that plays a significant role in the development and maintenance of cell-mediated immunity and central tolerance. The thymus is involved in the control of T lymphocyte maturation, positive and negative selection, and suppression of autoreactive T lymphocytes. Because the thymus is essential for the suppression of the immune response against autoantigens, it is not surprising to find that thymic tumors are associated with defects in thymic functions and with immunological disorders. Indeed, thymomas have been associated with a broad spectrum of autoimmune disorders, such as myasthenia gravis, pure red-cell aplasia, systemic lupus erythematosus and pemphigus vulgaris [1]. Renal involvement has been also reported, such as MCD, focal segmental glomerulosclerosis, membranous nephropathy and ANCA-associated glomerulonephritis [2]. Thymoma-associated glomerulonephritis is rare, as occurrence of nephropathy is rarely reported in most retrospective surgical series (< 1%). The most frequently described type of glomerular involvement is MCD [2], and it is thus considered as secondary MCD, as opposed to primary idiopathic MCD. There is no recommendation for treatment. However, the fundamental therapy is based on steroid therapy and thymectomy. The sensitivity of thymoma-associated MCD to steroids is usually about 84% [2]. Second line treatment is sometimes necessary and includes cyclosporine, cyclophosphamide, azathioprine, or chlorambucil, but severe adverse effects are possible and should be considered [2]. Recently, rituximab has been used for the treatment of primary MCD with good results and few adverse effects [3]. While rituximab has been successfully used in other autoimmune manifestations of thymoma [4], we did not find any report of its use in MCD associated with thymoma. We decided to treat this patient with rituximab instead of more conventional therapies (calcineurin inhibitors, cyclophosphamide) for several reasons. Firstly, to preserve kidney function and avoid nephrotoxicity related to some therapies. Secondly, because of the demonstrated efficacy of rituximab for idiopathic nephrotic syndrome caused by MCD [5] and finally for a strategy of “steroid saving” [6] . Here, we report the efficacy of rituximab in this pathology. In the present case, clinical symptoms disappeared within a few days, and remission was obtained within 3 weeks after administration of rituximab, without any adverse event.

The pathophysiological mechanisms involved in primary idiopathic and secondary MCD are not clearly identified. In primary idiopathic MCD, in addition to mechanisms disrupting the integrity of the cytoskeleton at the podocyte level, altered activation of T lymphocytes has also been suggested as a key factor [7]. Indeed T lymphocyte deregulation has been reported in vivo in patients and in animal models of MCD, with a lack of differentiation of T lymphocytes, a reduced number of T regulatory lymphocytes, increased secretion of both Th1 and Th2 cytokines and dysregulation of the cooperation between B and T lymphocytes [8, 9]. These abnormalities are also characteristic features of thymoma, thus suggesting that T-mediated immunologic disorders are important factors in the pathogenesis of MCD [10].

The efficacy of rituximab as a B-depleting agent in T cell-mediated affections such as MCD raises many questions about the mechanisms of action involved. Rituximab is known to have both direct and indirect effects. First, it is established that B lymphocytes are involved in modulation of T lymphocyte functions, such as activation, production of cytokines and growth factor synthesis. Therefore, depletion of B cells with rituximab will have an indirect effect on T cells by the disruption of T and B cooperation [11]. In addition, rituximab can act directly on the podocyte to maintain the cytoskeleton, after fixation on sphingomyelin phosphodiesterase acid-like 3B (SMPDL3B) [11]. In the case reported here, we did not find any modification of the cytokine environment, suggesting that the beneficial effect of rituximab may not rely on the modulation of lymphocyte activation.

In this present case, we report a beneficial effect of rituximab administration in thymoma-associated MCD. This case report does not allow us to draw any conclusions about the mechanism of action of rituximab, but we believe these findings argue for the safety and efficacy of rituximab use in thymoma-associated MCD, and therefore constitute a strong rationale for future studies.

## Data Availability

Not applicable.

## Abbreviations

MCD: Minimal Change Disease
CT: Computed Tomography
SMPDL3B: Sphingomyelin phosphodiesterase acid-like 3B
